# Rates of subacromial notching are low following reverse shoulder arthroplasty with a 135° inlay humeral component and a lateralized glenoid

**DOI:** 10.1016/j.jseint.2024.01.009

**Published:** 2024-02-10

**Authors:** Theresa Pak, Mariano E. Menendez, Reuben Gobezie, Benjamin W. Sears, Evan Lederman, Shoulder Arthroplasty Research Committee (ShARC) group, Anthony Romeo, Anup Shah, Asheesh Bedi, Bradford Parsons, Brandon Erickson, Bruce Miller, Christopher O’Grady, Daniel Davis, David Lutton, Joern Steinbeck, John Tokish, Julia Lee, Justin Griffin, Kevin Farmer, Matthew Provencher, Michael Bercik, Michael Kissenberth, Patric Raiss, Peter Habermeyer, Philipp Moroder, Robert Creighton, Russell Huffman, Sam Harmsen, Tim Lenters, Tyrrell Burrus, Tyler Brolin, Albert Lin, Brian C. Werner, Patrick J. Denard

**Affiliations:** a Center for Orthopedic Research and Education, Phoenix, AZ, USA; b Oregon Shoulder Institute, Medford, OR, USA; c Cleveland Shoulder Institute, Beachwood, OH, USA; d Western Orthopaedics, Denver, CO, USA; e University of Arizona College of Medicine – Phoenix, Phoenix, AZ, USA; f Shoulder Arthroplasty Research Committee, Naples, FL, USA; g University of Virginia Health System, Charlottesville, VA, USA

**Keywords:** Subacromial notching, Reverse shoulder arthroplasty, Abduction impingement, Lateralization, Inlay, Outcomes

## Abstract

**Background:**

Lateralization in reverse shoulder arthroplasty (RSA) decreases bony impingement and improves rotational range of motion, but has been theorized to increase the risk of subacromial notching (SaN). The purpose of this study was to evaluate the presence of SaN following RSA and its relationship with lateralization with a 135° inlay humeral component. The secondary purpose was to assess the association of SaN with functional outcomes.

**Methods:**

A retrospective review was performed from a multicenter prospectively collected database on patients who underwent primary RSA from 2015 to 2021. All RSAs were performed with a 135° inlay humeral component. SaN was defined as bony erosion with sclerotic margins on the undersurface of the acromion on final follow-up radiographs not present preoperatively. Postoperative implant positioning (inclination, distalization, and lateralization) were evaluated on minimum 1-year postoperative radiographs. Regression analyses were performed on implant and clinical variables to assess for risk factors. A separate analysis was performed to determine the association of SaN with clinical outcomes.

**Results:**

SaN was identified in 13 out of 442 shoulders (2.9%). Age, sex, body mass index, smoking status, diabetes mellitus, arm dominance had no relationship with SaN. Neither glenoid sided lateralization nor humeral offset were associated with SaN risk. Other implant characteristics such as distalization, glenosphere size, and postoperative inclination did not influence SaN risk. The presence of SaN did not affect patient-reported outcomes (American Shoulder and Elbow Surgeons: *P* = .357, Visual Analog Scale: *P* = .210) or range of motion.

**Conclusion:**

The rate of SaN is low and not associated with glenoid or humeral prosthetic lateralization when using a 135° inlay humeral component. When SaN occurs, it is not associated with functional outcomes or range of motion at short-term follow-up.

Lateralizing the center of rotation in reverse shoulder arthroplasty (RSA) has been reported to increase impingement-free rotational range of motion (ROM), improve rotator cuff tensioning, and enhance stability.^2^^,^^5^^,^^7^^,^^12^ Additionally, adduction impingement is a well-known consequence of a medialized RSA design^6^^,^^8^^,^^14^^,^^24^ and glenoid lateralization decreases scapular notching/adduction impingement.^15^^,^^21^

Biomechanical studies have raised concerns with lateralization and abduction impingement.^17^^,^^18^^,^^23^ The consequent subacromial notching (SaN) is an incompletely described phenomenon with limited clinical studies to date. One study with a 145° component and a medialized glenoid reported a 12.8% rate of SaN.^13^ However, this cohort was limited to an Asian population and a 36-mm glenosphere.

Therefore, the purpose of this study was to evaluate the presence of SaN following RSA and its relationship with glenoid-sided lateralization with a 135° inlay humeral component. The secondary purpose was to assess the presence of SaN on functional outcomes. We hypothesized that the SaN incidence would be low and not related to lateralization when utilizing a 135° inlay stem.

## Methods

### Database and study patients

A retrospective review was performed on a prospectively maintained multicenter database on patients who underwent primary RSA from 2015 to 2021. Inclusion criteria were as follows: minimum 1-year follow-up and primary RSA performed with a 135° inlay humeral component. Exclusion criteria were as follows: revision procedures, primary RSA for proximal humerus fractures, use of custom implants, and the presence of preoperative or postoperative acromial fracture. Institutional review board approval and patient consent was obtained before study inception as part of the prospective database enrollment.

### Surgical technique

RSAs were performed at 12 sites. In all cases, a deltopectoral approach was used with a 135° inlay humeral component (Univers Revers; Arthrex, Inc., Naples, FL, USA). For the glenoid, an anatomically shaped baseplate was used before 2018 (Universal Baseplate; Arthrex, Inc., Naples FL) and a modular circular baseplate (Modular Glenoid System; Arthrex, Inc., Naples, FL, USA) was used from 2018 to 2021. Glenoid-sided lateralization occurred through the baseplate and/or glenosphere and varied from 0 to 8 mm in 2 mm increments based on surgeon preference, patient anatomy, and soft-tissue tension. Humeral offset included the polyethylene liner and metallic spacer if used. Glenospheres with diameters ranging from 33 mm to 42 mm were implanted based on surgeon preference with a goal of matching to patient size and avoiding excessive anterior or posterior overhang. Subscapularis repair and postoperative rehabilitation were not standardized.

### Patient characteristics and outcome measures

Patient characteristics and patient-reported outcomes (PROs) were prospectively collected in a secure database. Baseline data collected included age, sex, body mass index (BMI), smoking status, history of diabetes mellitus, surgical side dominance. PROs and ROM were assessed at baseline and at the final follow-up. PROs obtained included American Shoulder and Elbow Surgeons (ASES) and Visual Analog Scale (VAS) scores. ROM was measured by the treating surgeon with a goniometer for active forward flexion, active external rotation (ER) in adduction (ER0), active ER with arm at 90° (ER90), and internal rotation (IR) with the arm at 90° (IR90). Internal rotation was also estimated to nearest spinal level (IR spine). Implant characteristics such as glenosphere size, glenoid-sided lateralization, and humeral offset were also recorded.

### Radiographic measurements

**Figure 1:**
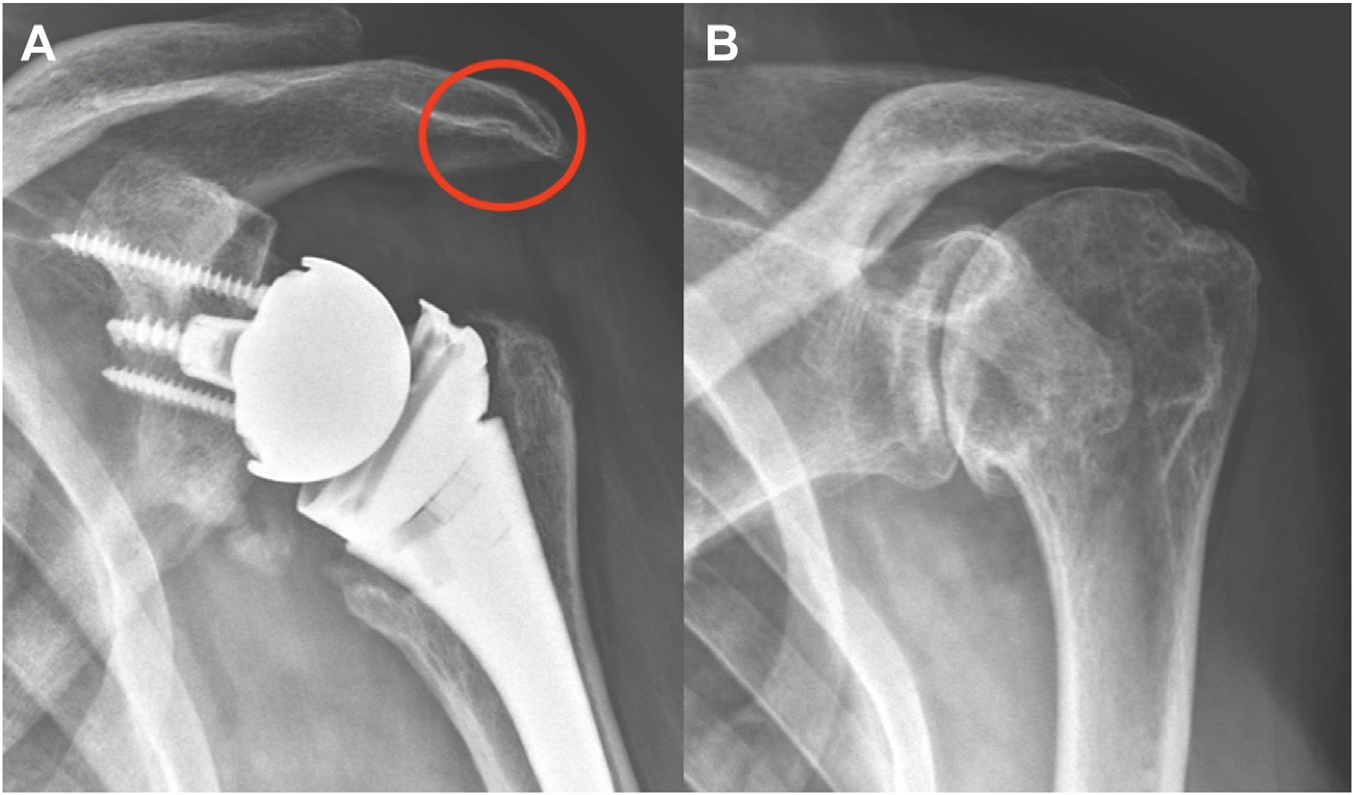
Grashey view of a left shoulder with subacromial notching as evidenced by bony erosion with sclerotic margins on the undersurface of the acromion (*circle*) at (**A**) final follow-up radiographs not present (**B**) preoperatively.

Preoperative, immediate postoperative and final follow-up radiographs were obtained including Grashey, axillary, and scapular Y views. Radiographs were reviewed by a fellowship-trained shoulder surgeon not involved in the surgeries (T.P.) in DICOM (digital imaging and communications in medicine) using Horos (Pixmeo, Bernex, Switzerland). SaN was defined as bony erosion with sclerotic margins on the undersurface of the acromion^13^ on final follow-up radiographs not observed on preoperative or immediate postoperative radiographs (Fig. 1). An independent examiner (T.P.) evaluated radiographs and any discrepancies were resolved by consensus with two senior authors (B.W., P.J.D.).

Measurements for postoperative inclination, distalization, and lateralization were made on postoperative radiographs. Glenoid inclination was determined by the ß angle as described by Maurer et al.^20^ Acromial humeral distance (AHD) was one metric used to determine distalization. The AHD was measured as described by Haidamous et al^11^ on Grashey views. To account for positioning of the arm, an initial line down the axis of the humerus was drawn. From there, intersecting lines from the undersurface of the acromion to the most superior aspect of the greater tuberosity were made.

**Figure 2:**
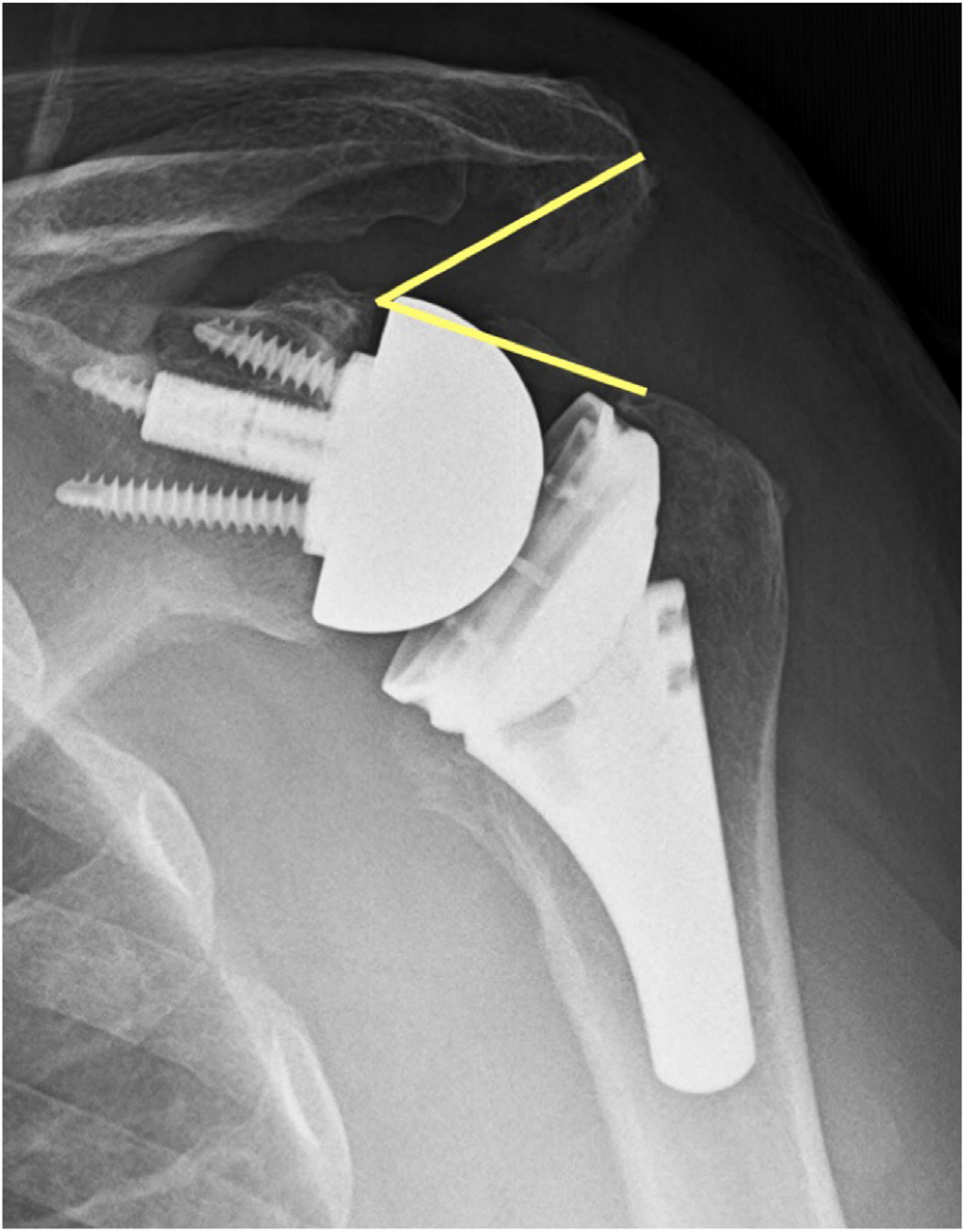
The DSA as described by Boutsiadis et al^3^ is made by drawing a line connecting 1) the lateral most aspect of the acromion and 2) the superior glenoid tubercle. From there, a second line is made from 2) the glenoid tubercle to 3) the superior most aspect of the greater tuberosity. A measurement between those 2 lines forms the DSA. *DSA*, distalization shoulder angle.

**Figure 3:**
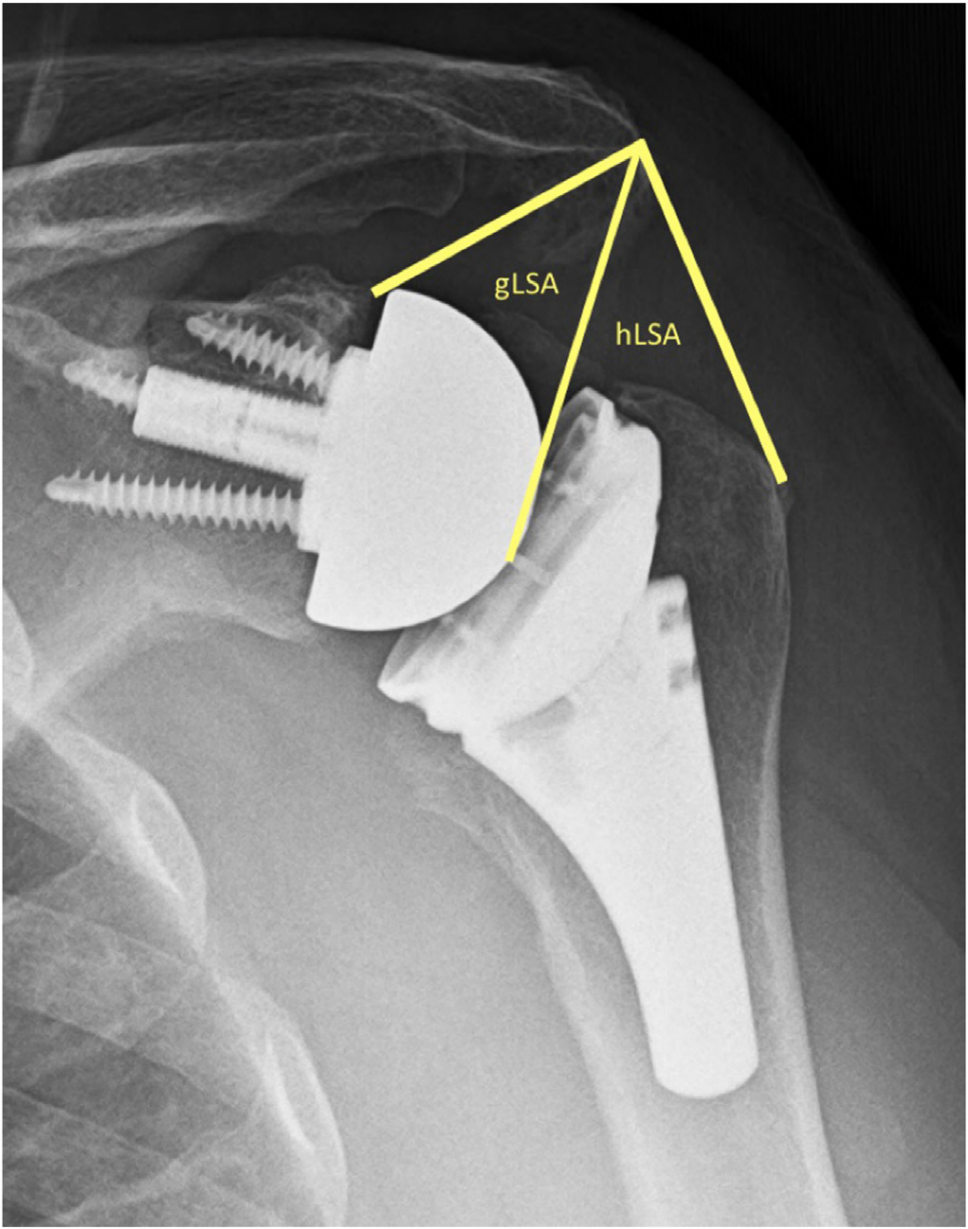
The LSA as described by Boutsiadis et al.^3^ A line connecting the 1) superior glenoid tubercle and 2) the lateral most aspect of the acromion is made. From there, a second line connects 2) the lateral most aspect of the acromion to 3) the lateral most aspect of the greater tuberosity. A measurement between those 2 lines forms the LSA. The LSA was further divided from the lateral most aspect of the glenophere to determine glenoid (gLSA) and humeral (hLSA) contributions. *LSA*, lateralization shoulder angle; *gLSA*, glenoid contribution to lateralization shoulder angle; *hLSA*, humeral contribution to lateralization shoulder angle.

Additional measurements for distalization and lateralization were also made according to the distalization shoulder angle and lateralization shoulder angle, respectively^3^ (Figs. 2 and 3). The lateralization shoulder angle was further divided into glenoid (gLSA) and humeral (hLSA) contributions at the most lateral point of the glenosphere as described by described by Schippers and Boileau^22^ (Fig. 3).

### Statistical analysis

All statistical analyses were performed in Statistical Package for the Social Sciences version 29 (IBM, Armonk, NY, USA). Evaluation of patient and implant risk factors for SaN was performed using a binary logistic regression analysis, with the presence of SaN as the dependent variable. For each independent variable, odds ratios, 95% confidence intervals and *P* values were generated. To assess any relationship between the presence of SaN on PROs and ROM, linear regression analyses were performed with the outcome of interest as the dependent variable, and SaN as well as numerous other potential confounders as independent variables. For each of these analyses, unstandardized beta coefficients, 95% confidence intervals and *P* values were generated for SaN.

## Results

A total of 442 RSAs met the study criteria. Baseline characteristics are summarized in Table 1. Glenoid metallic lateralization was 4 mm or larger in 88.9% of cases. Humeral lateralization was largely 6 mm or less (Table 2). SaN was observed in 13 patients, for an overall rate of 2.9%. Age, sex, BMI, history of diabetes mellitus, arm dominance had no relationship with SaN (Table 3).

**Table I:** Baseline patient characteristics.

Variable	
Patient demographics	Mean ± s.d.
Age (years)	70 ± 7.5
BMI	30.3 ± 6.4
	n = 442, %
Sex (male)	231, 52.3
DM	48, 10.9
Dominant arm	255, 57.7
PRO	Mean ± s.d.
ASES	39.9 ± 17.8
VAS	5.6 ± 2.5
ROM	
FF	93° ± 40
ER0	28° ± 20
ER90	24° ± 27
IR (spinal level)	L5 ± 3
IR90	18° ± 23

*BMI*, body mass index; *DM*, diabetes mellitus; *PRO*, patient-reported outcome; *ASES*, American Shoulder and Elbow Surgeons; *VAS*, visual analog scale; *ROM*, range of motion; *FF*, forward flexion; *ER0*, external rotation; *ER90*, external rotation with the arm at 90°; *IR*, internal rotation; *IR90*, internal rotation with the arm at 90°; *s.d.,* standard deviation.

**Table II:** Implant characteristics.

Glenoid lateralization (mm)	n	Humeral lateralization (mm)	n
0	38	3	250
2	11	6	119
4	184	9	36
6	130	12	17
8	79	15	14
		18	6

**Table III:** Regression results – subacromial notching risk.

Variable	OR (95% CI)	*P* value
Patient characteristics		
Age	0.06 (0.98-1.15)	.165
BMI	0.38 (0.96-1.13)	.369
Sex	−0.25 (0.13 to 4.69)	.789
DM	0.40 (0.29-7.61)	.630
Dominant arm	0.27 (0.41-4.21)	.646
Implant positioning and characteristics		
AHD	0.06 (0.33-3.47)	.915
DSA	−0.03 (0.90 to 1.05)	.465
gLSA	0.04 (0.93-1.17)	.459
hLSA	−0.07 (0.82 to 1.06)	.275
Glenoid metallic lateralization	0.02 (0.76-1.37)	.884
Humeral offset	−0.07 (0.73 to 1.19)	.561
Glenosphere size	−0.07 (0.66 to 1.32)	.681
ß angle	0.08 (0.96-1.23)	.184

*BMI*, body mass index; *DM*, diabetes mellitus; *AHD*, acromial humeral distance; *DSA*, distalization shoulder angle; *gLSA*, glenoid contribution to lateralization shoulder angle; *hLSA*, humeral contribution to lateralization shoulder angle; *OR*, odds ratio; *CI*, confidence interval.

Neither glenoid-sided lateralization as determined by metallic glenoid-sided lateralization and gLSA, nor humeral lateralization as determined by implant humeral offset and hLSA increased SaN risk. Distalization as determined by AHD and distalization shoulder angle was neither predictive nor protective of notching. Other implant characteristics such as glenosphere size or postoperative inclination did not have an effect on SaN risk (Table 3).

The presence of SaN did not affect PROs (ASES: *P* = .329, VAS: *P* = .191). There was no effect on ROM with the presence of SaN (FF: *P* = .763, ER0: *P* = .768, ER90: *P* = .595, IR spine: *P* = .880, IR90: *P* = .923) (Table 4).

**Table IV:** Regression results – presence of surbacromial notching.

Variable	B (95% CI)	*P* value
PRO		
ASES	4.49 (−4.55 to 13.50)	.329
VAS	−0.63 (−1.59 to 0.32)	.191
ROM		
FF	2.30 (−12.68 to 17.28)	.763
ER0	1.71 (−9.64 to 13.05)	.768
ER90	4.40 (−11.87 to 20.66)	.595
IR spine	0.14 (−1.63 to 1.90)	.880
IR90	−0.63 (−13.58 to 12.31)	.923

*PRO*, patient-reported outcome; *ASES*, American Shoulder and Elbow Surgeons; *VAS*, visual analog scale; *ROM*, range of motion; *FF*, forward flexion; *ER0*, external rotation; *ER90*, external rotation with the arm at 90°; *IR*, internal rotation; *IR90*, internal rotation with the arm at 90°; *CI*, confidence interval.

## Discussion

The main findings of this study were that the rate of SaN following RSA with a 135° inlay humeral component was low at 2.9%, and glenoid-sided lateralization even up to 8 mm did not increase SaN risk. Furthermore, the presence of SaN did not affect PROs or ROM.

SaN has largely been a theoretical concern with its description limited to biomechanical studies noting possible increase in acromial impingement with increased lateralization.^9^^,^^10^^,^^16^, ^17^, ^18^^,^^23^ Neither glenoid nor humeral sided lateralization conferred SaN risk (gLSA: *P* = .459, glenoid lateralization: *P* = .884, hLSA: *P* = .275, humeral offset: *P* = .561). However, in the one other clinical study on patients with a 12.8% rate of SaN, humeral lateralization was found to be a risk factor.^13^ In their series, a 36-mm glenosphere was implanted in all cases without additional lateralization, indicating that if any lateralization was introduced, it was originating from humeral offset. In contrast, humeral offset in the current study was primarily 6 mm or less with lateralization primarily being introduced from the glenoid. Given the comparably low SaN incidence of 2.9% in the present study, preferentially lateralizing through the glenoid may have a role in decreasing SaN.

The low SaN incidence in the present investigation may further be explained by racial morphologic differences and implant size mismatch. Prior studies have demonstrated morphologic differences around the shoulder girdle between East Asian and Western populations.^1^^,^^4^ Current implants may not accommodate the smaller morphometrics of the East Asian population.^4^ As mentioned, in their series of an Eastern Asian study population, a 36-mm glenosphere was implanted in all cases.^13^ In comparison, the current study utilized glenospheres with diameters from 33 mm to 42 mm with a goal of matching to patient size. The availability of smaller glenospheres may indicate an enhanced ability to decrease size mismatch, which has been demonstrated to affect outcomes.^19^ Further study in Asian populations with the use of smaller glenospheres may thus be warranted. Although precise guidelines on glenosphere size matching are currently lacking.

When SaN did occur, it did not affect 1 year ASES or VAS (*P* = .329, *P* = .191). ROM (FF, ER0, ER90, IR spine, IR90 - *P* > .05) was also not affected. With the exception of ASES score, this is largely consistent with what has been reported previously.^13^ As there are limited clinical data on SaN, further studies are needed to investigate the effect on functional outcomes. In the short term, however, SaN does not appear to impair function.

This study is not without its limitations including its retrospective nature. Secondly, given the very low incidence, patients with 1-year follow-up were included. However, as SaN has been up to this point a largely undescribed phenomenon, the inclusion of 1-year follow-up is justified. Third, although patient demographics such as age, sex, and BMI were included in the analysis, preoperative indication for RSA was not included. Additionally, it is possible the implant characteristics and patient demographics investigated may have an association with SaN; however, the low number of SaN cases introduces the potential for type II error in the findings. Finally, defining SaN remains unvalidated and this study was limited to use of plain radiographs. As such, preoperative films were scrutinized and compared to final follow-up films. All cases of SaN were reached by consensus.

## Conclusion

The rate of SaN is low and not associated with glenoid or humeral prosthetic lateralization when using a 135° inlay humeral component. When SaN occurs, it is not associated with functional outcomes or ROM at short-term follow-up.

## Disclaimers:

Funding: 10.13039/100007307Arthrex, Inc. Grant #: AIRR-00608-82.

Conflicts of interest: Dr. Patrick Denard reports Consultant, Royalties, and Research grants from Arthrex, Inc.; Stock ownership in PT Genie and Kaliber Labs.Dr. Gobezie reports Arthrex Inc.: paid consultat; he receives royalties and research funds. Dr. Benjamin W. Sears reports Consultant and Royalties – United Orthopaedic Corporation, Aeuvumed, Shoulder Innovations, BioPoly; Research funding/support – Arthrex, Inc., Exactech, Stryker, FX Solutions. Dr. Evan Lederman reports; Arthrex, Inc.: Consultant, Royalties, and Research support. Dr. Werner reports Arthrex, Inc.: Consultant, research support; Lifenet: Consultant; Biomet: Research support; Pacira: Research support. Dr. Menendez reports Arthrex, Inc: consultant. Dr. Anup Shah reports Arthrex, Inc.: Education/research consultant and Medacta: Royalties. Dr. Asheesh Bedi reports Arthrex, Inc.: Consultant and royalties. Dr. Bradford Parsons reports Arthrex, Inc.: Consultant and royalties; Editor JBJS reviews. Dr. Brandon Erickson reports AAOS: Board or committee member; American Orthopaedic Society for Sports Medicine: Board or committee member; American Shoulder and Elbow Surgeons: Board or committee member; Arthrex, Inc.: Paid consultant; Research support; DePuy, A Johnson & Johnson Company: Research support; Linvatec: Research support; PLOS One: Editorial or governing board; Smith & Nephew: Research support; Stryker: Research support. Dr. Bruce Miller reports AJSM: Editorial or governing board; Arthrex, Inc.: Paid consultant; FH Orthopedics: Royalties. Dr. Christopher O’Grady reports Education consultant/Speaker for: Arthrex, Inc., Stryker, Smith and Nephew, Mitek. Dr. Daniel Davis reports Arthrex, Inc.: Paid consultant, paid presenter/speaker; Catalyst OrthoScience: Stock or stock options; Board Member: Pennsylvania Orthopaedic Society; Board Member: Philadelphia Orthopaedic Society. Dr. David Lutton reports Arthrex, Inc.: Paid consultant, Paid speaker or presenter; Avanos: Paid speaker or presenter; CORE: Reviewer (not Editor). Dr. John Tokish reports Arthrex, Inc: IP royalties; Paid consultant; Paid presenter or speaker; Arthroscopy Association of North America: Board or committee member; Journal of Shoulder and Elbow Surgery: Editorial or governing board, financial or material support; Orthopedics Today: Editorial or governing board. Dr. Jorn Steinbeck reports Reviewer: JSES, JBJS; Arthrex, Inc.: Consultant. Dr. Julia Lee reports Arthrex, Inc.: Consulting and medical education; American Shoulder Elbow Society – Committee member. Dr. Kevin Farmer reports American Orthopaedic Society for Sports Medicine Florida Orthopaedic Society: Board or committee member; Arthrex, Inc. Paid consultant, paid presenter or speaker; Exactech, Inc.: Paid consultant. Dr. Mathew Provencher reports Royalties from Arthrex, Inc. Arthrosurface, Responsive Arthroscopy (2020), and Anika Therapeutics, Inc.; Consulting fees from Arthrex, Inc., Joint Restoration Foundation, Zimmer Biomet Holdings, and Arthrosurface; received grants from the Department of Defense, the National Institute of Health, and the DJO (2020); Honoria from Flexion Therapeutics; Editorial board or governing board member for SLACK, Inc.; Board or committee member for AANA, AAOS, AOSSM, ASES, SDSI, and SOMOs; serves on the medical board of trustees for the Musculoskeletal Transplant Foundation (through 2018). Dr. Michael Bercik reports American Shoulder and Elbow Surgeons: Board or committee member; Arthrex, Inc.: Paid consultant; WRS Specialists: Paid consultant. Dr. Michael Kissenberth reports Paid consultant for a company or supplier: Arthrex, Inc.; Financial or material support from a company or supplier: Hawkins Foundation; Board Member: Hawkins Foundation. Dr. Patric Raiss reports Paid consultant for Arthrex, Inc.; Shareholder Zurimed Technologies AG. Dr. Peter Habermeyer reports Royalties Arthrex, Inc. Dr. Philipp Moroder reports Consultant for Alyve Medical, Arthrex, and Medacta; Receives royalties from Alyve Medical, Arthrex, and Medacta. Dr. G. Russell Huffman reports Speakers bureau/paid presentations for a company or supplier: Arthrex, Inc., LIMA; Paid consultant for a company or supplier: Arthrex, Inc., LIMA; Stock or stock options in a company or supplier: Catalyst; Research support from a company or supplier as a PI: LIMA IDE. Dr. Samuel Harmsen reports Arthrex, Inc: Royalties; Paid consultant; Paid presenter or speaker; Research support; Embody, Inc.: Royalties; Paid consultant; Enovis, Inc.: Paid consultant; paid presenter; Genesis Software Innovations, LLC: Royalties; Stock or stock options; Shoulder Innovations, Inc: Royalties, Paid consultant; Paid presenter or speaker; Zimmer US Inc.; Paid consultant; Paid presenter or speaker; Stock or stock options. Dr. Tim Lenters reports Paid consultant: Arthrex, Inc.; Research support: Irispet; AAOS: social media committee. Dr. Matthew Tyrrell Burrus reports Arthrex, Inc.: Paid consultant; Paid presenter or speaker; Research support; Arthroscopy: Editorial or governing board. Dr. Tyler Brolin reports AAOS: Board or committee member; American Shoulder and Elbow Surgeons: Board or committee member; Arthrex, Inc.: IP royalties; Paid consultant; Research support; Elsevier: Publishing royalties, financial or material support; Orthofix, Inc.: Research support; Orthopedic Clinics of North America: Editorial or governing board; Zimmer: Research support. Dr. Romeo reports Arthrex Inc.: royalties, research support, paid consultant; Saunders/Mosby-Elsevier and SLAC Inc. publishing royalties and financial or material support; AANA and MLB: other financial or material support; Paragen Technologies: research support, stock or stock options.Dr. Creighton reports Arthrex: consultant; Arthrex, Breg and Smith & Nephew: research support. Dr. Griffin reports Arthrex inc: research support, royalties, paid consultant; Springer: publishing royalties. Dr. Lin reports Arthrex, Inc. and Tornier: paid consultant. The other author, her immediate family, and any research foundation with which she is affiliated have not received any financial payments or other benefits from any commercial entity related to the subject of this article.
